# Supplementary material for: Identification of novel, clinically correlated autoantigens in the monogenic autoimmune syndrome APS1 by proteome-wide PhIP-Seq
Source: eLife. 2020 May 15;9:e55053. doi: 10.7554/eLife.55053 (PMC7228772; doi:10.7554/eLife.55053)
Supplement: Supplementary file 1. — ND, nail dystrophy. HP, hypoparathyroidism. KC, keratoconjunctivitis. CMC, chronic mucocutaneous candidiasis. ID (D, C, B), Intestinal dysfunction (diarrheal-type, constipation-type, both). AIH, autoimmune hepatitis. POI, primary ovarian insufficiency. HTN, hypertension. HT, hypothyroidism. B12 def, B12 (vitamin) deficiency. DM, diabetes mellitus. SS, Sjogren’s-like syndrome. GH def, Growth hormone deficiency. AI, Adrenal Insufficiency. EH, (dental) enamel hypoplasia. TF, testicular failure. TIN, Tubulointerstitial Nephritis. Hpit, Hypopituitarism. UE, Urticarial eruption. D, Discovery cohort; V, Validation cohort. *Age at most recent evaluation [file elife-55053-supp1.docx]

**Supplementary File 1. APS1 cohort: Clinical Data.**

| **Patient Code** | **Gender** | **Age*** | **Clinical Phenotypes** | **Cohort** |
| --- | --- | --- | --- | --- |
| AIRE.04 | F | 14 | CMC, HP, AI, DM, EH, ND, HTN, SS, Pneumonitis, UE, GH def, ID (D) | **D** |
| AIRE.05 | F | 11 | CMC, HP, AI, AIH, Gastritis, EH, HTN, Pneumonitis, UE, Vitiligo, ID (B) | **D** |
| AIRE.09 | F | 10 | HP, AIH, EH, Pneumonitis, UE | **D** |
| AIRE.13 | F | 10 | CMC, HP, AI, Gastritis, UE, Vitiligo, ID (D) | **D** |
| AIRE.14 | M | 7 | CMC, AI, AIH, DM, Gastritis, EH, ND, KC, SS, Pneumonitis, UE, Vitiligo, B12 def, ID (C) | **D** |
| AIRE.18 | F | 18 | CMC, HP, AI, POI, ND, SS | **D** |
| AIRE.19 | M | 12 | CMC, HP, AI, AIH, Gastritis, EH, Pneumonitis, UE, GH def, Asplenia, ID (B) | **D** |
| AIRE.20 | F | 25 | CMC, AI, Gastritis, EH, KC, SS, ID (D) | **D** |
| AIRE.21 | M | 65 | CMC, HP, AI, HT, DM, EH, HTN, SS, Vitiligo, B12 def, ID (B) | **D** |
| AIRE.23 | M | 38 | CMC, HP, AI, Gastritis, TIN, EH, ND, KC, HTN, Vitiligo, Alopecia, B12 def, Asplenia, ID (D) | **D** |
| AIRE.24 | F | 15 | CMC, HP, AI, AIH, Gastritis, EH, KC, ID (C) | **D** |
| AIRE.27 | M | 18 | CMC, AI, AIH, DM, Gastritis, EH, KC, SS, Pneumonitis, UE, Vitiligo, B12 def, ID (B) | **D** |
| AIRE.08 | M | 11 | CMC, HP, AI, EH, UE | **D** |
| AIRE.07 | M | 12 | CMC, HP, AI, HT, ND, KC, Alopecia, ID (C) | **D** |
| AIRE.28 | M | 15 | CMC, HP, AI, Gastritis, EH, KC, Vitiligo, ID (C) | **D** |
| AIRE.22 | F | 7 | CMC, HP, AIH, Gastritis, EH, ND, SS, Pneumonitis, UE, Alopecia, GH def, ID (D) | **D** |
| AIRE.29 | M | 9 | AI, AIH, EH, ND, UE, Vitiligo, Alopecia, ID (B) | **D** |
| AIRE.30 | M | 17 | CMC, HP, AIH, EH, UE, Vitiligo, Alopecia, B12 def, ID (C) | **D** |
| AIRE.23c | F | 41 | CMC, HP, AI, HT, POI, EH, SS | **D** |
| AIRE.31 | F | 18 | CMC, HP, AI, AIH, DM, EH, ND, KC, SS, UE, Vitiligo, ID (D) | **D** |
| AIRE.33 | F | 14 | HP, AI, AIH, POI, EH, UE, ID (D) | **D** |
| AIRE.34 | F | 54 | CMC, HP, AI, HT, POI, Gastritis, EH, HTN, SS, Pneumonitis, B12 def, ID (D) | **D** |
| AIRE.35 | F | 23 | CMC, HP, AI, AIH, HT, POI, Gastritis, EH, SS, Pneumonitis, UE, B12 def, Asplenia, ID (D) | **D** |
| AIRE.11 | M | 19 | CMC, HP, AI, AIH, TF, Gastritis, EH, SS, UE, Vitiligo, GH def, ID (B) | **D** |
| AIRE.36 | M | 15 | HP, AI, EH, Alopecia | **D** |
| AIRE.37 | F | 28 | CMC, HP, AI, POI, EH, SS, UE, ID (D) | **D** |
| AIRE.38 | F | 7 | HP, AI, EH, ND, UE, Alopecia, ID (C) | **D** |
| AIRE.17 | F | 6 | CMC, HP, EH, KC, UE, ID (D) | **D** |
| AIRE.39 | F | 18 | CMC, HP, AI, AIH, HT, EH, ND, KC, Pneumonitis, UE, ID (B) | **D** |
| AIRE.40 | F | 16 | CMC, HP, AI, AIH, POI, EH, Pneumonitis, UE, Alopecia, Asplenia, ID (D) | **D** |
| AIRE.41 | M | 20 | CMC, AI, HT, TF, EH, HTN, Vitiligo, Alopecia, ID (D) | **D** |
| AIRE.44 | F | 24 | CMC, HP, AI, POI, Gastritis, EH, ND, KC, HTN, SS, UE, Alopecia, B12 def, ID (D) | **D** |
| AIRE.46 | F | 22 | CMC, HP, AI, EH, KC, SS, B12 def, GH def, ID (B) | **D** |
| AIRE.12 | F | 7 | CMC, HP, AI, Gastritis, EH, KC, SS, Pneumonitis, UE, Vitiligo, B12 def, ID (D) | **D** |
| AIRE.06 | F | 16 | CMC, HP, AI, AIH, HT, Gastritis, EH, HTN, SS, Vitiligo, ID (D) | **D** |
| AIRE.50 | F | 26 | CMC, AI, Gastritis, HTN, SS, Pneumonitis, UE, B12 def, ID (B) | **D** |
| AIRE.02 | M | 51 | CMC, HP, AI, TF, Gastritis, EH, HTN, SS, Hpit, Pneumonitis, Vitiligo, B12 def, ID (D) | **D** |
| AIRE.03 | F | 19 | HP, AI, POI, TIN, EH, HTN, Pneumonitis, UE | **D** |
| AIRE.52 | F | 9 | HP, HT, EH, UE, Vitiligo, ID (D) | **D** |
| AIRE.53 | F | 8 | CMC, HP, AI, HT, EH, HTN, UE, ID (D) | **V** |
| AIRE.58 | M | 16 | CMC, HP, AI, TF, Gastritis, EH, ND, KC, Alopecia, B12 def, ID (D) | **V** |
| AIRE.59 | M | 7 | CMC, HP, AI, EH, ND, Alopecia, ID (D) | **V** |
| AIRE.60 | M | 19 | CMC, ND, EH, Alopecia, ID (C) | **V** |
| AIRE.61 | F | 54 | CMC, HP, AI, EH, SS, Pneumonitis, ID (C) | **V** |
| AIRE.62 | F | 15 | AI, AIH, HT, Gastritis, Pneumonitis, UE, ID (C) | **V** |
| AIRE.55 | M | 19 | CMC, HP, AI, Gastritis, EH, UE, Alopecia | **V** |
| AIRE.69 | M | 18 | CMC, AI, AIH, ID (B) | **V** |
| AIRE.56 | M | 2 | AIH, EH, UE, ID (D) | **V** |
| AIRE.54 | F | 7 | CMC, HP, AI, EH, Pneumonitis, UE | **V** |
| AIRE.63 | F | 15 | CMC, HP, AI, EH, B12 def, ID (B) | **V** |
| AIRE.71 | F | 30 | CMC, HP, Gastritis, EH, Pneumonitis, Vitiligo, ID (D) | **V** |
| AIRE.71B | M | 15 | CMC, AI, HT, Gastritis, ND, Pneumonitis, Alopecia | **V** |
| AIRE.74 | F | 11 | CMC, HP, AI, HT, Gastritis, TIN, EH, SS, Pneumonitis, UE, Alopecia, ID (C) | **V** |
| AIRE.68 | F | 15 | CMC, AI, Gastritis, EH, SS, Pneumonitis, B12 def, ID (C) | **V** |
| AIRE.70 | F | 16 | CMC, SS, UE, B12 def, ID (C) | **V** |
| AIRE.66 | M | 13 | CMC, HP, AI, DM, EH, UE, Alopecia | **V** |
| AIRE.67 | M | 20 | CMC, HP, AI, Pneumonitis, UE, Vitiligo, ID (D) | **V** |
| AIRE.87 | F | 15 | CMC, HP, AI, AIH, HT, EH, Pneumonitis, Vitiligo, B12 def, ID (B) | **V** |
| AIRE.65C | M | 2 | CMC, HP, AI, UE, ID (C) | **V** |
| AIRE.65B | M | 6 | CMC, HP, AI, EH | **V** |
| AIRE.65 | F | 11 | CMC, HP, AI, EH, UE, Vitiligo, GH def, ID (D) | **V** |
| AIRE.73 | F | 13 | CMC, HP, AIH, HT, POI, EH, ID (B) | **V** |
| AIRE.76 | M | 10 | CMC, HP, UE, Vitiligo, ID (D) | **V** |
| AIRE.86 | F | 3 | HP, UE, ID (C) | **V** |
| AIRE.77 | M | 10 | HP, AIH, HT, SS, Pneumonitis, Vitiligo, Alopecia, ID (D) | **V** |
| AIRE.78 | M | 2 | HP | **V** |
| AIRE.79 | M | 10 | CMC, HP, AI, AIH, EH, UE, GH def, Asplenia | **V** |
