# Supplementary material for: Identification of novel, clinically correlated autoantigens in the monogenic autoimmune syndrome APS1 by proteome-wide PhIP-Seq
Source: eLife. 2020 May 15;9:e55053. doi: 10.7554/eLife.55053 (PMC7228772; doi:10.7554/eLife.55053)
Supplement: Supplementary file 2. — D, Discovery cohort; V, Validation cohort. [file elife-55053-supp2.docx]

**Supplementary File 2. Non-APS1 control cohort: Clinical Data.**

| **Non-APS1 Control Code** | **Gender** | **Age** | **Cohort** |
| --- | --- | --- | --- |
| A2 | M | 21 | V |
| A3 | F | 22 | V |
| A5 | M | 22 | V |
| A6 | F | 23 | V |
| A7 | M | 23 | V |
| A8 | M | 23 | V |
| A9 | F | 24 | V |
| B2 | M | 25 | V |
| B3 | M | 25 | V |
| B5 | F | 26 | V |
| B6 | F | 27 | V |
| B7 | M | 28 | V |
| B8 | F | 29 | V |
| B9 | F | 31 | V |
| C2 | F | 33 | V |
| C3 | M | 33 | V |
| C5 | M | 34 | V |
| C6 | F | 35 | V |
| C7 | M | 35 | V |
| C8 | F | 36 | V |
| C9 | F | 36 | V |
| D2 | M | 38 | V |
| D3 | M | 39 | V |
| D5 | M | 41 | V |
| D6 | M | 42 | V |
| D7 | F | 43 | V |
| D8 | F | 45 | V |
| D9 | M | 45 | V |
| E1 | F | 46 | V |
| E2 | F | 47 | V |
| E3 | M | 47 | V |
| E4 | F | 48 | V |
| E5 | F | 48 | V |
| E6 | M | 48 | V |
| E7 | M | 48 | V |
| E8 | M | 48 | V |
| E9 | M | 48 | V |
| F1 | F | 50 | V |
| F2 | F | 50 | V |
| F3 | F | 51 | V |
| F4 | F | 51 | V |
| F5 | F | 51 | V |
| F6 | M | 51 | V |
| F7 | F | 52 | V |
| F8 | M | 52 | V |
| F9 | M | 52 | V |
| G1 | M | 55 | V |
| G2 | M | 55 | V |
| G3 | M | 55 | V |
| G4 | M | 56 | V |
| G5 | M | 57 | V |
| G6 | M | 57 | V |
| G8 | F | 59 | V |
| G9 | F | 60 | V |
| H1 | M | 61 | V |
| H2 | M | 61 | V |
| H3 | F | 62 | V |
| H4 | F | 63 | V |
| H5 | F | 63 | V |
| H6 | M | 63 | V |
| H8 | F | 65 | V |
| NC-30 | n/a | n/a | D (PhIP-Seq) |
| NC-31 | n/a | n/a | D (PhIP-Seq) |
| NC-45 | n/a | n/a | D (PhIP-Seq) |
| NC-46 | n/a | n/a | D (PhIP-Seq) |
| NC-47 | n/a | n/a | D (PhIP-Seq) |
| NC-48 | n/a | n/a | D (PhIP-Seq) |
| NC-49 | n/a | n/a | D (PhIP-Seq) |
| NC-50 | n/a | n/a | D (PhIP-Seq) |
| NC-51 | n/a | n/a | D (PhIP-Seq) |
| NC-53 | n/a | n/a | D (PhIP-Seq) |
| NC-54 | n/a | n/a | D (PhIP-Seq) |
| NC-55 | n/a | n/a | D (PhIP-Seq) |
| NC-56 | n/a | n/a | D (PhIP-Seq) |
| NC-57 | n/a | n/a | D (PhIP-Seq) |
| NC-58 | n/a | n/a | D (PhIP-Seq) |
| NC-59 | n/a | n/a | D (PhIP-Seq) |
| NC-60 | n/a | n/a | D (PhIP-Seq) |
| NC-61 | n/a | n/a | D (PhIP-Seq) |
| NC-62 | n/a | n/a | D (PhIP-Seq) |
| NC-64 | n/a | n/a | D (PhIP-Seq) |
| NC-65 | n/a | n/a | D (PhIP-Seq) |
| NC-66 | n/a | n/a | D (PhIP-Seq) |
| NC-67 | n/a | n/a | D (PhIP-Seq) |
| NC-68 | n/a | n/a | D (PhIP-Seq) |
| NC-69 | n/a | n/a | D (PhIP-Seq) |
| NC-70 | n/a | n/a | D (PhIP-Seq) |
| NC-72 | n/a | n/a | D (PhIP-Seq) |
| NC-77 | n/a | n/a | D (PhIP-Seq) |

D, Discovery cohort; V, Validation cohort.
