# Supplementary material for: Identification of novel, clinically correlated autoantigens in the monogenic autoimmune syndrome APS1 by proteome-wide PhIP-Seq
Source: eLife. 2020 May 15;9:e55053. doi: 10.7554/eLife.55053 (PMC7228772; doi:10.7554/eLife.55053)
Supplement: Supplementary file 3. [file elife-55053-supp3.docx]

**Supplementary File 3. Tissue-restricted expression patterns of validated and putative novel APS1 antigens.**

| Orthogonally validated | | |
| --- | --- | --- |
| **Gene (Human/mouse)** | **Protein Atlas: RNA specificity category (Tissue)**^14^ | **Selected literature annotations** |
| *RFX6/Rfx6* | Tissue enhanced | Pancreas  Islets (Piccand et al., 2014; S. B. Smith et al., 2010)  Intestine  Enteroendocrine cells (Gehart et al., 2019; Piccand et al., 2019; S. B. Smith et al., 2010) |
| *KHDC3L/Khdc3* | Group enriched | Ovary  Oocytes (Y. Zhang et al., 2018; K. Zhu et al., 2015) |
| *ACP4/Acp4* | Tissue enhanced | Testes (Yousef et al., 2001)  Dental enamel (Green et al., 2019; Seymen et al., 2016; C. E. Smith et al., 2017) |
| *ASMT/Asmt* | Tissue enhanced | Brain  Pineal Gland (Rath et al., 2016) |
| *GIP/Gip* | Tissue enriched | Intestine  Enteroendocrine cells (Moody et al., 1984) |
| *NKX6-3 / Nkx6-3* | Group enriched | Pancreas  PP-cells (Schaum et al., 2018)  Intestine (Alanentalo et al., 2006) |
| *PDX1 / Pdx1* | Group enriched | Pancreas  Islets (Holland et al., 2002; Stoffers et al., 1997) |

| Putative / non-validated | |
| --- | --- |
| **Gene (Human/mouse)** | **Protein Atlas: RNA specificity category (Tissue)**^14^ |
| *BNIP1* | Low tissue specificity |
| *RASIP1* | Low tissue specificity |
| *RNF165* | Tissue enhanced (brain) |
| *TBATA* | Tissue enhanced (testis) |
| *TCOF1* | Low tissue specificity |
| *PNO1* | Low tissue specificity |
| *CDK5R1* | Tissue enriched (brain) |
| *FAM47A* | Tissue enriched (testis) |
| *SPEF2* | Low tissue specificity |
| *SNRPD1* | Low tissue specificity |
| *RBMXL3* | Tissue enriched (testis) |
| *RBMXL2* | Tissue enriched (testis) |
| *PDYN* | Tissue enriched (brain) |
| *CYSRT1* | Tissue enriched (esophagus) |
| *SARNP* | Low tissue specificity |
| *PRR11* | Tissue enhanced (lymphoid tissue) |
| *AP5B1* | Tissue enhanced (bone marrow) |
| *ANGPTL8* | Tissue enriched (liver) |
| *NOP2* | Low tissue specificity |
| *CRH* | Tissue enriched (placenta) |
| *NOG* | Tissue enhanced |
| *SAMD4A* | Tissue enhanced |
| *SRRT* | Low tissue specificity |
| *CATSPER2* | Tissue enriched (testis) |
| *DAZ4* | Group enriched (stomach, testis) |
| *DAZ2* | Group enriched (stomach, testis) |
| *DAZ1* | Group enriched (stomach, testis) |
| *ARFRP1* | Low tissue specificity |
| *C7orf50* | Low tissue specificity |
| *C3orf30* | Tissue enriched (testis) |
| *RFX4* | Group enriched (brain, testis) |
| *CAMK2N1* | Tissue enhanced (brain) |
| *ZNF367* | Tissue enhanced (bone marrow, lymphoid tissue) |
| *ZNF439* | Low tissue specificity |
| *POU1F1* | Tissue enriched (pituitary gland) |
| *HAPLN1* | Tissue enriched (placenta) |
| *MUC5B* | Tissue enhanced (gallbladder, lymphoid tissue, salivary gland) |
| *SRSF8* | Low tissue specificity |
| *ARRDC3* | Low tissue specificity |
| *PLAGL2* | Low tissue specificity |
| *CDK2* | Low tissue specificity |
| *NOP16* | Low tissue specificity |
| *DEDD2* | Tissue enhanced (vagina) |
| *SUGP2* | Low tissue specificity |
| *STK19* | Low tissue specificity |
| *SAYSD1* | Tissue enhanced (testis) |
| *BCL9L* | Low tissue specificity |
| *GET4* | Low tissue specificity |
| *FCMR* | Group enriched (blood, intestine, lymphoid tissue) |
| *MORC2* | Tissue enhanced (testis) |
| *TRIM74* | Tissue enhanced (stomach) |
| *TRIM50* | Group enriched (pancreas, stomach) |
| *POLDIP3* | Low tissue specificity |
| *CROCC2* | Tissue enhanced (brain, epididymis, fallopian tube) |
| *NBPF19* | Low tissue specificity |
| *SOX8* | Tissue enriched (brain) |
| *SLC18A1* | Tissue enhanced (adrenal gland, intestine) |
| *QSER1* | Low tissue specificity |
| *H1F0* | Tissue enhanced (bone marrow) |
| *ZNF618* | Low tissue specificity |
| *AOAH* | Tissue enhanced (blood, lymphoid tissue) |
| *SLX4IP* | Low tissue specificity |
| *NANOG* | Tissue enhanced (blood, testis) |
