# Supplementary material for: Identification of novel, clinically correlated autoantigens in the monogenic autoimmune syndrome APS1 by proteome-wide PhIP-Seq
Source: eLife. 2020 May 15;9:e55053. doi: 10.7554/eLife.55053 (PMC7228772; doi:10.7554/eLife.55053)
Supplement: Supplementary file 4. [file elife-55053-supp4.docx]

**Supplementary File 4. Antibody information by application.**

| antibody | Application  (IF: immunofluorescence;  RLBA: radioligand binding assay; CBA: cell-based assay) | dilution |
| --- | --- | --- |
| Anti-NLRP5 (Santa Cruz, Dallas, TX; #sc-50630) | NLRP5 RLBA | 1:50 |
| Anti-SOX10 (Abcam, Cambridge, MA, #ab181466) | SOX10 RLBA | 1:25 |
| Anti-RFX6 (R&D Systems, Minneapolis, MN; #AF7780) | RFX6 RLBA | 1:50 |
| Anti-KHDC3L (Abcam, #ab170298) | KHDC3L RLBA | 1:25 |
| Anti-CYP11A1 (Abcam, #ab175408) | CYP11A1 RLBA | 1:50 |
| Anti-NKX6-3 (Biorbyt, Cambridge, Cambridgeshire, UK; #orb127108) | NKX6-3 RLBA | 1:50 |
| Anti-GIP (Abcam, #ab30679) | GIP RLBA | 1:50 |
| Anti-PDX1 (Invitrogen, Carlsbad, CA, #PA5-78024) | PDX1 RLBA | 1:50 |
| Anti-ASMT (Invitrogen, #PA5-24721) | ASMT RLBA | 1:25 |
| Anti-CHGA (Abcam, Cambridge, MA, USA, # ab15160) | Tissue IF | 1:5000 |
| Human serum | Tissue IF  CBA IF  RLBA | 1:4000 (Tissue)  1:500 (CBA)  1:25 (RLBA) |
| **Secondary abs:**  488 goat anti-human IgG (Life Technologies, Waltham, MA, USA: #A11013)  546 goat anti-rabbit IgG (Life Technologies, A11010) | Tissue IF | 1:400 |
| **Secondary abs:**  647 goat anti-human IgG (Thermo Fisher, #A-21445)  488 goat anti-rabbit IgG (Thermo Fisher, #A-11034) | CBA IF | 1:1000 |
| Anti-DYKDDDDK (D6W5B) (Cell Signaling Technologies, Danvers, MA; #14793) | CBA IF;  ACP4 RLBA;  TPH1 RLBA | 1:2000 (CBA IF)  1:125 (RLBA) |
| **Nuclear staining:**  Hoechst dye (Invitrogen, #33342)  DAPI (Thermo Fisher, #D1306) | Tissue IF  CBA IF |  |
